# Intra-Tumoral Activation of Endosomal TLR Pathways Reveals a Distinct Role for TLR3 Agonist Dependent Type-1 Interferons in Shaping the Tumor Immune Microenvironment 

**DOI:** 10.3389/fonc.2021.711673

**Published:** 2021-07-26

**Authors:** Graham Thomas, Luca Micci, Wenjing Yang, Joseph Katakowski, Cecilia Oderup, Purnima Sundar, Xiao Wang, Kenneth G. Geles, Shobha Potluri, Shahram Salek-Ardakani

**Affiliations:** Cancer Immunology Discovery, Worldwide Research, Development and Medical, Pfizer Inc., San Diego, CA, United States

**Keywords:** TLR - toll-like receptor, TLR3 agonist, TLR7 agonist, TLR9 agonist, IFN - interferon, tumor, scRNA-Seq

## Abstract

Toll-like receptor (TLR) agonists have received considerable attention as therapeutic targets for cancer immunotherapy owing to their ability to convert immunosuppressive tumor microenvironments towards a more T-cell inflamed phenotype. However, TLRs differ in their cell expression profiles and intracellular signaling pathways, raising the possibility that distinct TLRs differentially influence the tumor immune microenvironment. Using single-cell RNA-sequencing, we address this by comparing the tumor immune composition of B16F10 melanoma following treatment with agonists of TLR3, TLR7, and TLR9. Marked differences are observed between treatments, including decreased tumor-associated macrophages upon TLR7 agonist treatment. A biased type-1 interferon signature is elicited upon TLR3 agonist treatment as opposed to a type-2 interferon signature with TLR9 agonists. TLR3 stimulation was associated with increased macrophage antigen presentation gene expression and decreased expression of PD-L1 and the inhibitory receptors *Pirb* and *Pilra* on infiltrating monocytes. Furthermore, in contrast to TLR7 and TLR9 agonists, TLR3 stimulation ablated FoxP3 positive CD4 T cells and elicited a distinct CD8 T cell activation phenotype highlighting the potential for distinct synergies between TLR agonists and combination therapy agents.

## Introduction

Toll-like receptors (TLRs) represent a first-line in host defense, providing a means by which signals derived from invading pathogens or host insult initiate activation of the innate immune system (1). Activation of macrophages and dendritic cells through TLRs elicits inflammatory cytokine production, upregulates antigen presentation machinery, and instructs dendritic cells (DCs) to migrate to tissue draining lymph nodes, initiating adaptive immune processes (2). In mice and humans, the TLR family of receptors is represented by 10 and 12 members, respectively. These distinct receptors differ in their ligand selectivity, subcellular localization, and cell subset distribution, enabling the detection of a diverse range of insults. Besides TLR3, mouse and human TLRs all interact with the adapter protein MyD88 leading to nuclear factor-κB (NF-κB) dependent expression of inflammatory cytokines. Additional TLR-induced signaling pathways proceed *via* the TRIF, TRAM, and TIRAP adapter proteins, ultimately leading to interferon (IFN) regulatory factor (IRF) and NF-κB dependent gene expression (3, 4). Thus, stimulation of distinct TLRs induces overlapping gene expression profiles, yet notable differences do exist between receptors (5).

The immune-stimulatory properties of TLRs have led to their exploration as cancer immunotherapy agents (6) and FDA approvals have been granted for several TLR agonists in oncology. These include the BCG vaccine for non-metastatic bladder cancer whose effects are largely mediated *via* TLR2/4 (7), and the TLR7 agonist imiquimod which is approved for the treatment of acidic keratosis and basal cell carcinoma (8). Many other agonists targeting TLRs have been considered for use as clinical oncology agents, including agonists targeting TLR2, TLR3, TLR7, TLR8, and TLR9, see (9) and (6) and references within. Single-agent trials with these compounds have typically shown modest signs of clinical efficacy mimicking findings from the pre-clinical setting. Reasons for their incomplete clinical benefit include dose-limiting toxicity and the requirement for intra-tumoral delivery (9). Furthermore, as TLR agonists engage innate immunity, the absence of adaptive immune de-repression of cytotoxic T-cells may mask potential efficacy. Indeed, the rationale that TLR agonists will polarize the tumor microenvironment from an unfavorable ‘cold’ state to an immune-stimulatory ‘hot’ state, providing synergy with checkpoint inhibitors that influence adaptive immunity has led to the inception of numerous clinical trials combining these agents (6, 10, 11).

More needs to be done to understand the immunomodulatory role of individual TLRs in the tumor, and the influence of TLR agonists on the immune microenvironment. An improved understanding in this area may aid in the design of rational combinations of TLR agonists with additional immunotherapies. Here we treat B16F10 melanoma with agonists of the endosomal TLRs TLR3, TLR7 and TLR9 to understand the influence of these agents on immune polarization in a ‘cold’ tumor model. Transcriptomic profiling using single-cell RNA-Seq unveils considerable differences in the tumor-immune microenvironment between TLR agonists. Most notably, we identify the presence of a type-1 interferon dominated gene signature and the absence of regulatory CD4+ Foxp3+ Tregs in response to TLR3 stimulation. These effects may be attributed both to the DC selective-expression of TLR3 and its unique intracellular signaling characteristics.

## Materials and Methods

### Animals

Six- to eight-week-old female C57BL/6 mice were purchased from Jackson Laboratories (Bar Harbor, ME). All animals were housed in a pathogen-free vivarium facility at Rinat/Pfizer Inc (South San Francisco, CA), and experiments were conducted according to protocols in accordance with the Institutional Animal Care and Use Committee (IACUC) guidelines.

### Cells

B16F10 melanoma cells were cultured in Dulbecco’s Modified Eagle Medium supplemented with 10% fetal bovine serum and 100 IU/mL penicillin-streptomycin at 37°C in an atmosphere of 5% carbon dioxide and IMPACT tested for pathogens at the Research Animal Diagnostic Laboratory (Columbia, MO). Pathogen-free cells in the exponential growth phase were harvested and used for tumor inoculation.

### Subcutaneous Tumor Models in Mice

C57BL/6 mice were inoculated subcutaneously with 5 × 10^5^ B16F10 cells in 0.1 mL of phosphate-buffered saline (PBS). Three animals were recruited into each treatment arm 10 days post-inoculation, at which point tumors volume were approximately 150 mm^3^. Tumor size was measured in two dimensions using a digital caliper. The volume was expressed in cubic millimeters using the formula V= 0.5 x (L x W^2^), where L and W are the long and short diameters of the tumor, respectively. 50 µL of each TLR agonist was delivered by intratumoral injection in PBS, or PBS vehicle control was used. The following agonists were used in this study: TLR3 agonist Poly I:C (100 µg total, Invivogen, CA), a TLR7/8 agonist (150 µg total) lacking the C18 lipid moiety (12) and referred here within as TLR7 due to the known inactivity of murine TLR8, and TLR9 agonist CpG1826 (13) (100 µg total).

### Single-Cell RNA-Seq and Gene Expression Quantification

Harvested tumors were dissociated to obtain single-cell suspensions using the mouse tumor dissociation kit (Miltenyi Biotec; Bergisch Gladbach, Germany) according to the manufacturer’s protocol and cells from three individual mouse tumors pooled for each TLR treatment condition. Cells were counted using a Vi-CELL (Beckman Coulter; Brea, CA), and stained using fluorescently labeled anti-mouse CD45 antibody (clone 30F11, Thermo Fisher Scientific; Waltham, MA) to allow purification of live CD45^+^ cells using a FACSAria II cell sorter (BD Biosciences; San Jose, CA). Purified CD45^+^ cells were counted using the Cellometer K2 Viability Cell Counter (Nexcelom; Lawrence, MA) before to loading on a Chromium Single Cell Chip (10x Genomics; Pleasanton, CA) per manufacturer’s guidelines. Library construction was performed using 50 ng cDNA following the Chromium Single Cell 5’ Library and Gel Bead Kit protocol (10x Genomics). Libraries were sequenced using the NovoSeq 6000 platform (Illumina; San Diego, CA). Data were processed using the Cell Ranger v2.1.1 (10x Genomics) to generate count-level data for further analysis. Each lane of cells was processed independently using the Cell Ranger count. The unique molecular identifier (UMI) counts for each sample were then merged using Seurat v2.3.1, requiring that the number of expressed genes for each cell was > 500 and < 5000. Cells with > 5% of UMI originated from mitochondrial genes were removed. Genes expressed in at least three cells were kept and then normalized and scaled using the default setting in Seurat. Raw sequence data relating to this study have been deposited at Gene Expression Omnibus under accession number GSE179449.

### Single-Cell Clustering, Annotation, Differential Expression, and GSEA

For each sample, we selected the top 2000 highly variable genes (HVG). These genes were combined into 3,756 HVG for downstream analysis. Canonical correlation analysis (CCA) (14) was then performed to align cells across different samples using the top 20 CCA components. Cell clustering was performed on the aligned CCA space using Seurat. In total 16 cell clusters were generated at resolution 1.2. The cell identity was determined by a manual review of top differentially expressed genes in each cell cluster. To identify genes that were differentially regulated upon TLR treatment, we performed differential gene expression (DGE) analysis between treatment conditions within clusters using the Wilcoxon rank-sum test. Functional enrichment of DE genes within each cluster was performed using fGSEA by considering the ranked gene lists generated in the comparison between TLR treatment arm and vehicle control. Visualization of single-cell RNA-Seq data was performed using Seurat, or in R using ggplot2 and heatmap.2.

## Results

### Single-Cell RNA-Seq Defines the Immune Infiltrate of B16F10 Melanoma

**Figure 1:**
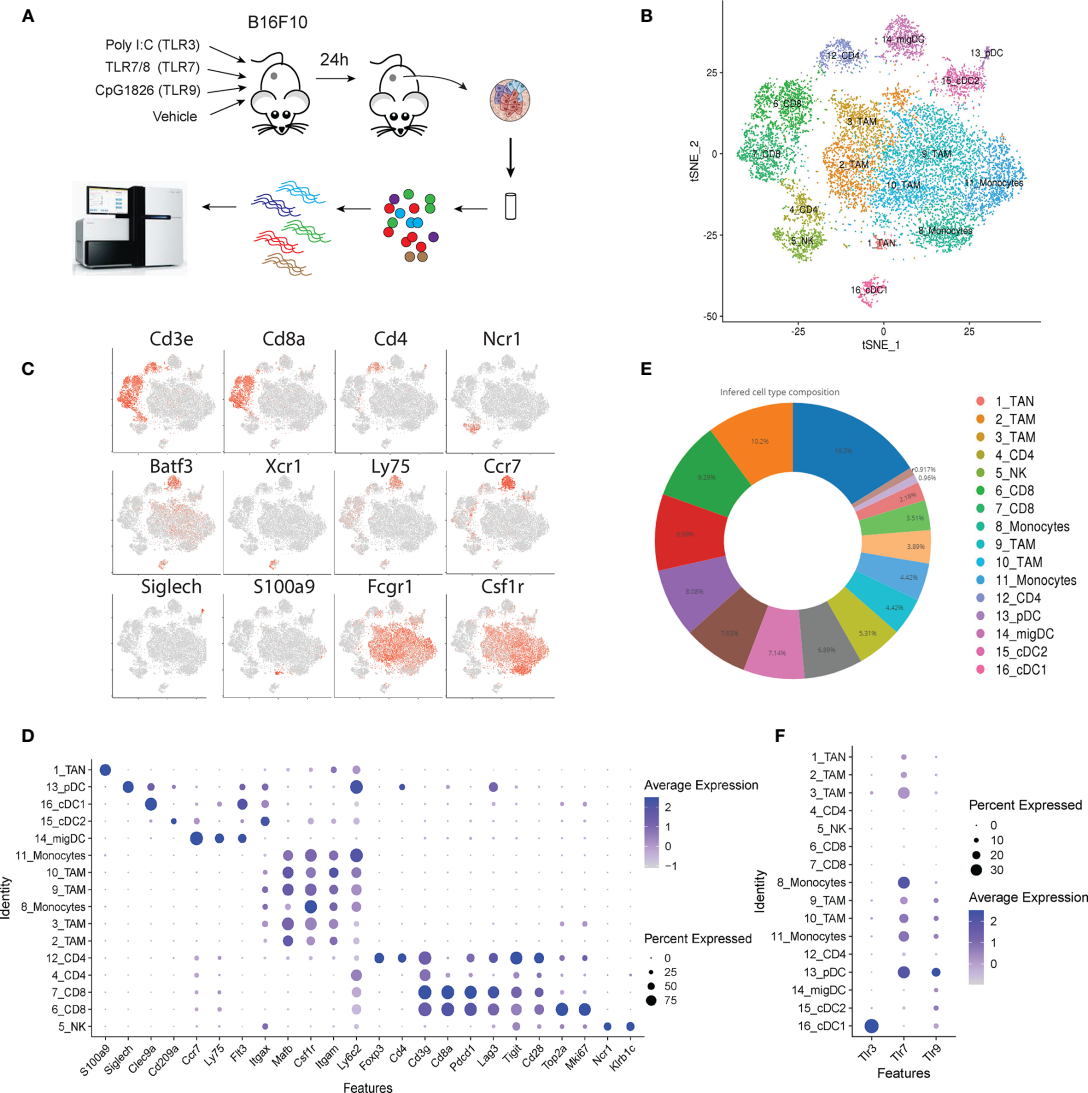
Schematic overview of experiment. **(A)** B16F10 tumors were grown to ~150 mm^3^ and intra-tumorally injected with TLR agonists. 24 hours post injection tumors were harvested and prepared for scRNA-Seq to profile tumor immune landscape following treatment. **(B)** tSNE plot showing the presence of tumor associated immune cell subsets identified by scRNA-Seq. **(C)** Gene expression intensities for key lineage marker genes plotted on tSNE biaxial plot demarcating major T cell (*CD3e, CD4, CD8a*), NK cell (*Ncr1*) dendritic cell (*Batf3, Xcr1, Ly75, Ccr7, Siglech*) neutrophil (*S100a9*) and macrophage (*Fcgr1, Csf1r*) subsets. **(D)** Dot plot showing expression distribution of a wider panel of phenotypic markers for major immune cell subsets identified in **(C)**. **(E)** Pie chart showing the frequency distribution between cells in clusters identified in **(B)** across all tumor samples. **(F)** Dot plot showing the expression of *Tlr3*, *Tlr7* and *Tlr9* genes in tumor-infiltrating CD45^+^ cells.

To assess the influence of TLR agonists on tumor immune activation profiles, B16F10 melanoma was chosen as a poorly immunogenic ‘cold’ tumor model to understand whether different TLR agonists differentially affect the tumor immune microenvironment. We focused our analysis at 24 hours post-treatment to understand the effects of TLR treatment on myeloid and DC polarization and gain insight into potential downstream processes that occur as a result of this activation. Subcutaneous B16F10 melanoma tumors were grown to 150 mm^3^ then injected I.T. with selective agonists targeting TLR3, TLR7or TLR9, or vehicle control respectively (**Figure 1A**). Twenty-four hours post-treatment CD45^+^ cells were FACS purified from three independent biological replicates, pooled and processed for 10x single-cell RNA-Sequencing. mRNA profiles belonging to 3,756 cells spanning all four conditions were clustered, defining 16 tumor-associated immune cell subsets (**Figure 1B**). Tumor-associated myeloid populations were identified including one tumor-associated *S100a9* positive neutrophil population (TAN); six macrophage (TAM)/monocyte populations expressing *Csf1r*, CD64 (*Fcgr1*) and CD11b (*Itgam*); one cDC1 population expressing *Batf3* and *Clec9a*; one cDC2 population marked by *Cd209a* expression, a migratory DC subset expressing *Ccr7* and *Ly75* and a *Siglech* positive pDC population (**Figures 1C, D**). Additionally, two CD8 T cell populations were identified that express activation and exhaustion-associated markers including *Pdcd1, Lag3, Tim3* and *Tigit*, these were found to differ in their proliferation signatures as determined by differential *Mki67* and *Top2a* expression. One NK cell population expressing *Ncr1* was present alongside one CD4 T cell population co-expressing *Cd4* and *Foxp3*; finally another CD3 positive T cell population putatively classified as CD4 positive yet possessing low CD4, somewhat higher CD8 expression and higher levels of *Cd7* and may thus be naïve CD8 T cells (15). Myeloid cells, consisting of tumor-associated monocytes and macrophages, represented 58.7% of the total tumor immune infiltrate and were by far the most abundant component of the immune cell infiltrate. CD4 and CD8 T cells accounted for 7.4% and 15.7%, respectively, NK cells represented 4.4%, and dendritic cells comprised 12.8% (**Figure 1E**). To identify populations that respond to TLR stimulation within the tumor microenvironment, we assessed TLR3, 7 and 9 expression within clusters (**Figure 1F**). TLR expression was restricted to myeloid populations with very low levels observed in NK and T cell subsets. Within the myeloid compartment expression profiles were found to be notably distinct. TLR3 was restricted to the classical dendritic cell (cDC) population *Xcr1^+^* cDC1s, whereas TLR7 was highly expressed across TAM populations and pDCs, but less abundant in cDCs. TLR9 was generally less abundant than TLR7, however, it was observed in pDCs, both DC1 and DC2 subsets, and across monocyte and macrophage subsets 9, 10, and 11. Thus, the tumor immune landscape is characterized by abundant tumor-associated myeloid populations that differentially express TLRs and the major adaptive and innate cytotoxic lymphocyte subsets.

### Characterization of Intra-Tumoral TLR Expressing Cell Subsets

**Figure 2:**
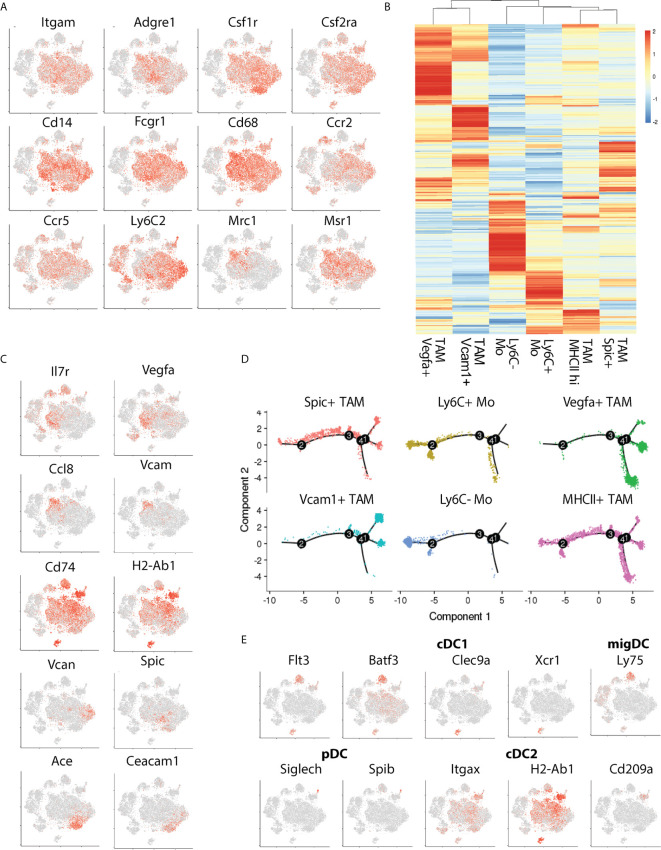
Transcriptional phenotyping of tumor-associated macrophage populations. **(A)** The expression of canonical macrophage lineage markers denotes a large cluster of TAMs comprising the bulk of the CD45^+^ infiltrate, **(B)** Hierarchical clustering of TAM subset marker genes identified during Seurat subset clustering, **(C)** Expression distribution of TAM-subset specific marker genes, **(D)** Pseudotime plot showing relative distances of TAMs to one another, split by cluster, denoting transcriptional relationships between TAM subsets, and, **(E)** tSNE plot showing marker distribution of key cDC-subset associated genes.

To better understand the characteristics of the TLR expressing monocyte and macrophage populations we surveyed the expression profiles of prototypical (**Figure 2A**) and subset-specific marker genes (**Figure 2B** and **Supplemental Table 1**) for the four TAM (clusters 2, 3, 9 and 10) and two monocyte clusters (8 and 11). All subsets expressed high levels of *Cd11b*, *Csf1r, Csf2ra, Cd14, Cd64* and *Cd68* confirming their initial classification as tumor-associated macrophages and monocytes (**Figure 2A**). The relative expression of *Adrge1* (F4/80) and *Cd206* were higher in TAM clusters 2 and 3, implying that these are mature TAMs. Phenotypic clustering identified markers that distinguished TAM populations (**Figure 2C**), cluster 2 was classified by expression of *Vegfa* and *Il7r* while *Vcam* and *Ccl8* (MCP-3) demarcated cluster 3 (**Figure 2C**). TAM cluster 9 possessed abundant MHCII-associated gene expression, including H2-Ab1 and *Cd74* suggesting that these cells may have a role in CD4 T cell activation. Myeloid cluster 10 expressed few unique genes but did express the transcription factor *Spic* alongside monocyte cluster 8, which we defined as nonclassical (*Ly6c* ^-^) monocytes based on previously defined profiles that include selective *Ceacam*1 and *Ace* expression (16). Finally, monocyte cluster 11 were determined to be classical (*Ly6c^+^*) inflammatory monocytes based upon *Vcan* and *Ly6C2* expression (17). To explore relationships between these TAMs, we used single-cell trajectory analysis (**Figure 2D**). Classical and nonclassical monocytes were adjacent, while *Vegfa* and *Vcam1* positive TAMs were distal to the monocytes. MHCII^+^ and *Spic* ^+^ TAMs occupied an intermediate branch of the trajectory tree. We also identified intra-tumoral DC subsets, including two *Flt3^+^, Batf3^+^* cDC1 populations that differentially express Dec205 *(Ly75)* and *Clec9a*, pDCs expressing *Siglech* and *Spib*, and the CD11b positive cDC2 population expressing *Cd209a* and high levels of MHCII (**Figure 2E**). These findings establish the presence of multiple discreet DC and TAM populations possessing distinct differentiation states that may respond differently to TLR stimulation.

### Tlr3 Agonist Elicits a Type 1 Interferon Signature Within the Intratumoral Myeloid Compartment

**Figure 3:**
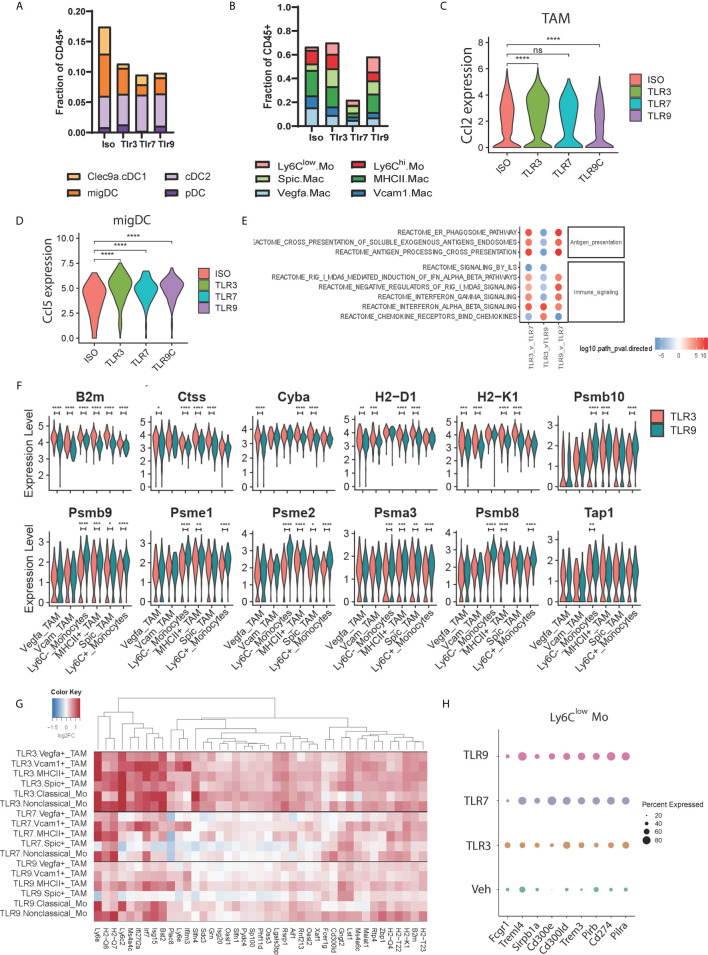
Functional profiling of TAM populations during TLR treatment. **(A, B)** The frequency of tumor-associated **(A)** dendritic cell and **(B)** macrophage subsets amongst CD45^+^ cells 24 hours post injection of TLR agonist, **(C, D)** Violin plots showing the expression distribution of **(C)** *Ccl2* mRNA between treatments for all TAM subsets, and **(D)** *Ccl5* mRNA within the activated migDC subset. **(E)** fGSEA pathway enrichment of Reactome pathways of TAM-associated genes compared to vehicle control, **(F)** Violin plots showing expression distribution of antigen-presentation associated genes in TAM subsets for TLR3-treated (red) and TLR9-treated (blue) tumors, **(G)** Hierarchical clustering of differentially expressed genes upregulated upon TLR3-treatment (clusters 5 and 11 from **Figure S1E**) showing uniform induction of gene expression in non-TLR3 expressing TAM subsets, and **(H)** Dotplot showing the relative expression of differentially expressed IgG and TNF receptor family members in nonclassical monocytes upon TLR agonist treatment. Statistical p values for panels **(C, D, F)** derived from Wilcoxon test; n.s., not shown, *p <= 0.05, **p <= 0.01, ***p <= 0.001, ****p <= 0.0001.

To address whether distinct TLR agonists impart similar effects on the tumor microenvironment, we assessed the responses of individual TAM and DC subsets to TLR treatment. First, we assessed changes in cell frequencies within the DC compartment. TLR3, 7 and 9 agonists moderately decreased DC frequencies to a similar degree, however, the relative abundance of individual subsets was not affected potentially reflecting DC activation and migration to draining lymph nodes (**Figure 3A**). However, within the TAM compartment, we found that TLR7 agonism led to a profound reduction in TAM frequencies 24h post-treatment (**Figure 3B**), while TLR3 and TLR9 stimulation had little impact on overall numbers. Instead, TLR3 and TLR9 agonists altered TAM subset composition, with both treatments decreasing in the number of *Vegfa* and *Vcam1* positive macrophages (**Figure 3B**). This reduction was offset by a relative increase in Ly6C^low^ monocytes by 3.4 and 4.8-fold for TLR3 and TLR9 and 2.8 and 2.0 for *Spic* positive macrophages. In order to understand potential chemoattractants contributing to this recruitment we evaluated the expression of *Ccl2* and *Ccl5*. We found that TLR3 agonist increased, while TLR9 agonist decreased *Ccl2* expression in TAM and that DCs did not express this chemokine at an appreciable level (**Figures 3C** and **S1A**). *Ccl5* was expressed at a low level by TAMs (**Figure S1B**) but highly by DCs, specifically we found that the activated migDC subset expressed high levels of *Ccl5* in response to treatment with all TLR agonists (**Figures 3D** and **S1B**). Thus, TLR agonists elicit *Ccl2* and *Ccl5* expression within the myeloid compartment, potentially underpinning the recruitment of monocytes following TLR agonist treatment.

To understand differences in TAM gene expression resulting from TLR agonist treatment we combined TAM subsets to obtain an aggregated view of TLR treatment. Pathway analysis identified differences in antigen presentation, immune signaling pathways, and chemokine signaling between treatment conditions (**Figure 3E**). Relative to TLR7, TLR3 and TLR9 agonists both increased antigen presentation gene expression (**Figure 3E**). Consistent with this TLR3 and TLR9 agonists enhanced interferon pathway utilization, however, while TLR3 increased type-1 interferon pathway (IFNα/β) genes, TLR9 possessed a type-2 interferon (IFNγ) biased gene signature (**Figure 3E**). At the individual TAM subset level, we found that TLR3 and TLR9 agonists both upregulated antigen-presentation genes compared to vehicle control (**Figures S1C, D**). TLR3 stimulation showed relatively higher expression of genes involved in MHC-I antigen presentation, including *B2m*, *Tap1*, *H2-D1* and *H2-K1* compared to TLR9. At the same time TLR9 demonstrated higher expression of intracellular antigen processing apparatus, including immunoproteasome components *Psmb8/9/10* and *Psme1/2* (**Figure 3F**). Furthermore, TLR3 agonist increased antigen presentation-associated gene expression across TAM subsets, whereas the effects of TLR9-dependent gene expression were biased towards myeloid populations expressing higher levels of TLR9, notably clusters 9, 10 and 11.

The relatively uniform induction of antigen presentation genes across TAM subsets by TLR3 agonist contrasted with the restricted expression of TLR3 by cDC1 subset 16 (**Figure 1F**). We questioned whether this was representative of TLR3-induced gene expression and if TLR3-dependent effects differ from TLR7 and TLR9 induced changes in TAM gene expression. To address this, we performed differential expression analysis between TLR ligand and vehicle control-treated samples for each TAM cluster and evaluated patterns of differential gene expression by hierarchical clustering (**Figures 3G** and **S1E**). TLR3 agonist-induced gene expression was generally consistent across TAM subsets, supporting a model of indirect activation. Increased MHC-I antigen presentation genes and IFNα/β pathway utilization are consistent with a role of DC1-derived type 1 interferon in regulating TAM gene expression. Consistent with its broad expression, TLR7 agonist widely affected gene expression, albeit impacting an overlapping, but distinct gene set to the TLR3 agonist poly I:C. TLR9 stimulation was found to influence similar genes to TLR7 (**Figure 3G**). However, induced expression levels in TLR9 high classical and nonclassical monocyte subsets were higher than those in TLR9 low *Vegfa* and *Vcam1* positive TAMs (**Figure 3G**). Finally, we assessed differential gene expression amongst members of the TNF and IgG gene families as critical gene modulators of immune activation. Multiple genes were differentially regulated (**Figure S1F**), and expression typically trended in the same direction relative to vehicle control independent of agonist treatment. Surprisingly however, nonclassical monocyte cluster 8 demonstrated a unique expression pattern upon TLR7 and TLR9 treatment (**Figure 3H**) characterized by lower levels of *Fcgr1* and higher levels of the inhibitory receptors Cd274 (PD-L1), *Pirb* and *Pilra* as well higher expression of *Trem3, Treml4, Cd300ld, Cd300e.* These data indicate that the main effects of TLR7 and TLR9-dependent gene expression occur in the TME *via* interactions with their cognate TLR and involve the upregulation of inhibitory receptors on nonclassical monocytes. TLR3-dependent gene expression however, appears to be independent of TLR expression and may result indirectly from interferon IFNα/β secreted by TLR3 expressing cDC1 cells.

### TLR3 Agonist Decreases Signature of CD4+ Regulatory T Cells in the TME

**Figure 4:**
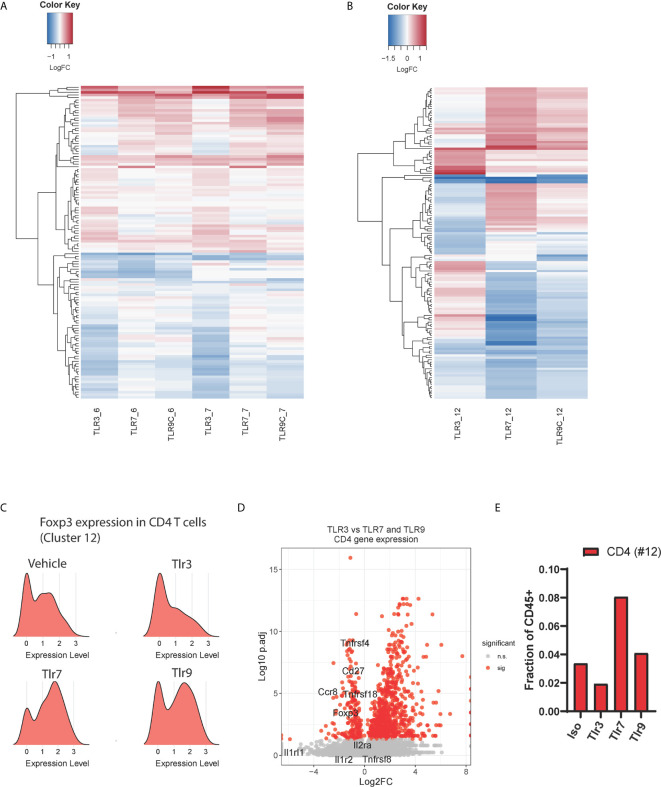
Effects of TLR treatment on adaptive immune cell populations. Hierarchical clustering of differential expressed genes between treatment conditions for, **(A)** CD8 T cell subsets 6 (activated, non-proliferative) and 7 (activated, proliferative), and **(B)** CD4 T cell subset 12. **(C)** Ridge plot showing *Foxp3* mRNA expression in CD4 T cell cluster 12 upon TLR agonist treatment, **(D)** The relative expression of Treg-associated genes in CD4 T cells (cluster 12) from TLR3-treated samples compared to the average expression of these genes in the same cluster for TLR7 and TLR9-treated samples, and **(E)** the total frequency of tumor-infiltrating CD4 T cells as a percentage of CD45+ cells upon treatment with TLR agonists.

Type-1 interferons profoundly influence adaptive immune responses by regulating antigen presentation thus influencing the activation and survival of effector T cells (18). We hypothesized that the TLR3-induced IFNα/β response distinctly affected the adaptive immune compartment to that induced by TLR7 and TLR9 agonists. As MHCI presents antigen to CD8 T cells, we first assessed changes in CD8 T cell gene expression. Hierarchical clustering of differentially regulated CD8 T cell genes showed that TLR3 agonist does indeed impart a unique transcriptional signature (**Figure 4A**). All TLR ligands induced gene signatures consistent with early T cell activation indicated by enhanced cell cycle pathway utilization (**Figures S2A, B**). However, TLR3 treatment selectively increased gene expression terms associated with translational and ribosome utilization. Changes in T cell phenotypes at this 24-hour timepoint are not consistent with *de novo* priming and trafficking of naïve cells from the lymph node to the tumor. Rather we expect these distinct transcriptional signatures reflect differences in the quality of TCR engagement mediated by myeloid-T cell interactions within the tumor upon treatment with these distinct TLR agonists.

Similar effects of TLR3 were observed within the CD4 T cell compartment (cluster 12), TLR3 stimulation with Poly I:C led to a distinct gene expression profile characterized by increased type-1 interferon signaling associated genes including *Isg15*, *Irf7* and *Bst2* (**Figures 4B** and **S2C**). As TLR-induced inflammatory cytokines are known to induce regulatory T cell (Treg) expansion (19), we questioned whether this could be explained by altered Treg polarization. Strikingly, we found that *Foxp3* expression was considerably lower in TLR3- treated CD4 T cells than in vehicle control. In contrast, TLR7 and TLR9 agonists both upregulated *Foxp3* expression on CD4 T cells (**Figure 4C**). To affirm this observation, we plotted the expression of tumor-specific Treg signature genes (20) comparing TLR3 gene expression against TLR7 and TLR9 induced gene expression in CD4 T cell cluster 12 (**Figure 4D**). These results show lower expression of Treg signature genes upon TLR3 stimulation 24h post treatment, consistent with a relative reduction in the number of Foxp3+ regulatory CD4 T cells upon TLR3 stimulation (**Figure 4E**). Thus, TLR3 treatment leads to a unique intra-tumor T cell phenotype characterized by distinct CD8 RNA expression profile and the loss of a regulatory T cell gene signature 24 hours post-treatment.

## Discussion

We set out to compare the influence of intra-tumoral TLR agonist delivery on tumor immune composition. Because TLR agonists provide essential co-stimulation to the innate immune system, they can convert the TME from ‘cold’ to ‘hot’, making these compelling candidates for cancer immunotherapy. To our surprise, we observed substantial differences in immune response profiles to treatment with distinct TLR agonists targeting TLR3, TLR7 and TLR9. These effects were most evident within the tumor-associated macrophage compartment, likely due to their relatively high TLR expression. TAM subsets were found to differ in the absolute and relative expression of distinct TLRs, which also signal *via* distinct pathways. TLR7 and TLR9 were found to be more broadly expressed, and both commonly elicit the MyD88-NF-κB axis, whereas TLR3 expression was restricted to the cDC1 population and signals *via* TRIF-IRF pathway (3).

Most notably, we observed that TLR7 drove a profound loss of TAMs 24 hours post-treatment, which was not observed for TLR9 despite their redundant intracellular signaling pathways. As TLR7 is more abundant than TLR9, this difference may be explained by receptor expression. However, distinct ligand properties may also influence downstream TLR biology (21). TLR3 and TLR9 agonists decreased the frequency of mature TAMs expressing F4/80 and CD206, leading to a concomitant increase in monocyte frequencies. The transient loss of mature macrophages is a common feature of acute inflammation and has been postulated to allow infiltrating inflammatory monocytes to differentiate and orchestrate appropriate inflammatory responses (22). In this regard, TLR3 and TLR9 agonists increased mRNA expression of chemokines *Ccl2* and *Ccl5* within intra tumoral myeloid populations that may influence subsequent cell recruitment and activation.

Increased TAM MHCI expression and signatures of early CD8 activation were a common feature of TLR agonist treatment. However, TLR7 and TLR9 agonists both elicited a parallel increase in *Foxp3* positive CD4 T cells, one explanation for which could be TAM derived IL-10 produced because of Myd88-NF-κB signaling (23, 24). TLR3-dependent effects mediated by Poly I:C did not possess this Treg signature at the 24 hr timepoint yet did maintain abundant antigen presentation and early CD8 T cell activation gene signatures. TLR3 treatment coincided with a type 1 interferon pathway gene signature, which was observed broadly within the tumor immune microenvironment. As cDC1 cells most abundantly express TLR3 and were found to be the only major source of TLR3 within the tumor, our findings are consistent with DC-derived type 1 interferon-mediated polarization of the tumor immune microenvironment. An alternative interpretation is that poly I:C elicits these broad effects through the ubiquitously expressed intracellular RNA-sensing RigI-Mda5 pathway rather than indirectly *via* TLR3-IFNα/β pathway (25). Indeed, our pathway analysis identified a RigI-Mda5 gene signature upon poly I:C treatment (**Figure 3C**). However, the genes contributing to the RigI-Mda5 pathway signature overlap with type 1 interferon pathway signature genes. Furthermore, our pathway analysis identified a stronger association between the RigI-Mda5 pathway and the TLR9 agonist CpG, which is not a ligand for either the RigI or Mda5 receptors (26), arguing that the RigI-Mda5 association may be a false positive. Finally, we did not observe TLR3, TLR7 or TLR9 expression in previously published RNA-Seq data of *in vitro* cultured B16F10 melanoma (data not shown), ruling our direct effects of agonist treatment on the tumor itself (27). Ultimately our data demonstrate that the TLR3 agonist poly I:C elicits fundamentally distinct immunologic effects compared to TLR7 and TLR9 agonists.

Here, using a system-wide interrogation of the tumor immune microenvironment following treatment with three distinct TLR agonists, we demonstrate that three distinct tumor immunophenotypes are elicited within the TAM compartment. It should be noted that one limitation of our study is that these effects are only reported for a single tumor model and at a single timepoint. Additional studies will be necessary to determine the extent to which these observations translate into different models. As cancer immunotherapy approaches frequently rely upon combinations (6, 28), these findings highlight the importance of appreciating the broader implications of agents’ effects on the tumor immune microenvironment in order to ensure such combinations are both rational and complementary.

## Data Availability Statement

The datasets presented in this study can be found in online repositories. The names of the repository/repositories and accession number(s) can be found below: https://www.ncbi.nlm.nih.gov/geo/, GSE179449.

## Ethics Statement

Mice were maintained and all animal experiments were conducted according to the protocols approved by the Institutional Animal Care and Use Committee of CID Pfizer (South San Francisco) and Worldwide Research and Development (La Jolla), Pfizer Inc.

## Author Contributions

Conception and design: LM, CO, SP, and SS-A. Development of methodology: LM, CO, and PS. Acquisition of data: LM, CO, and PS. Analysis and interpretation of data: GT, WY, XW, LM, CO, JK, KG, and SS-A. Writing, review, and/or revision of the manuscript: GT and SS-A. All authors contributed to the article and approved the submitted version.

## Funding

Funding was provided by Pfizer, Inc.

## Conflict of Interest

All authors were employed by Pfizer Inc. Pfizer employees may hold stock/stock options in the company.

## Publisher’s Note

All claims expressed in this article are solely those of the authors and do not necessarily represent those of their affiliated organizations, or those of the publisher, the editors and the reviewers. Any product that may be evaluated in this article, or claim that may be made by its manufacturer, is not guaranteed or endorsed by the publisher.

## References

[1] Takeda K Kaisho T Akira S. Toll-Like Receptors. Annu Rev Immunol (2003) 21:335–76. 10.1146/annurev.immunol.21.120601.141126 12524386

[2] Watts C West MA Zaru R. TLR Signalling Regulated Antigen Presentation in Dendritic Cells. Curr Opin Immunol (2010) 22(1):124–30. 10.1016/j.coi.2009.12.005 20083398

[3] Takeda K Akira S. Toll-Like Receptors. Curr Protoc Immunol (2015) 109:14 2 1–0. 10.1002/0471142735.im1412s109 25845562

[4] Kawasaki T Kawai T. Toll-Like Receptor Signaling Pathways. Front Immunol (2014) 5:461. 10.3389/fimmu.2014.00461 25309543 PMC4174766

[5] Huang Q Liu D Majewski P Schulte LC Korn JM Young RA. The Plasticity of Dendritic Cell Responses to Pathogens and Their Components. Science (2001) 294(5543):870–5. 10.1126/science.294.5543.870 11679675

[6] Smith M Garcia-Martinez E Pitter MR Fucikova J Spisek R Zitvogel L. Trial Watch: Toll-Like Receptor Agonists in Cancer Immunotherapy. Oncoimmunology (2018) 7(12):e1526250. 10.1080/2162402X.2018.1526250 30524908 PMC6279325

[7] Heldwein KA Liang MD Andresen TK Thomas KE Marty AM Cuesta N. TLR2 and TLR4 Serve Distinct Roles in the Host Immune Response Against Mycobacterium Bovis BCG. J Leukoc Biol (2003) 74(2):277–86. 10.1189/jlb.0103026 12885945

[8] Vacchelli E Galluzzi L Eggermont A Fridman WH Galon J Sautes-Fridman C. Trial Watch: FDA-Approved Toll-Like Receptor Agonists for Cancer Therapy. Oncoimmunology (2012) 1(6):894–907. 10.4161/onci.20931 23162757 PMC3489745

[9] Iribarren K Bloy N Buque A Cremer I Eggermont A Fridman WH. Trial Watch: Immunostimulation With Toll-Like Receptor Agonists in Cancer Therapy. Oncoimmunology (2016) 5(3):e1088631. 10.1080/2162402X.2015.1088631 27141345 PMC4839356

[10] Frega G Wu Q Le Naour J Vacchelli E Galluzzi L Kroemer G. Trial Watch: Experimental TLR7/TLR8 Agonists for Oncological Indications. Oncoimmunology (2020) 9(1):1796002. 10.1080/2162402X.2020.1796002 32934889 PMC7466852

[11] Le Naour J Galluzzi L Zitvogel L Kroemer G Vacchelli E. Trial Watch: TLR3 Agonists in Cancer Therapy. Oncoimmunology (2020) 9(1):1771143. 10.1080/2162402X.2020.1771143 32934877 PMC7466857

[12] Smirnov D Schmidt JJ Capecchi JT Wightman PD. Vaccine Adjuvant Activity of 3M-052: An Imidazoquinoline Designed for Local Activity Without Systemic Cytokine Induction. Vaccine (2011) 29(33):5434–42. 10.1016/j.vaccine.2011.05.061 21641953

[13] Ballas ZK Krieg AM Warren T Rasmussen W Davis HL Waldschmidt M. Divergent Therapeutic and Immunologic Effects of Oligodeoxynucleotides With Distinct CpG Motifs. J Immunol (2001) 167(9):4878–86. 10.4049/jimmunol.167.9.4878 11673492

[14] Butler A Hoffman P Smibert P Papalexi E Satija R. Integrating Single-Cell Transcriptomic Data Across Different Conditions, Technologies, and Species. Nat Biotechnol (2018) 36(5):411–20. 10.1038/nbt.4096 PMC6700744 29608179

[15] Aandahl EM Sandberg JK Beckerman KP Tasken K Moretto WJ Nixon DF. CD7 Is a Differentiation Marker That Identifies Multiple CD8 T Cell Effector Subsets. J Immunol (2003) 170(5):2349–55. 10.4049/jimmunol.170.5.2349 12594257

[16] Mildner A Schonheit J Giladi A David E Lara-Astiaso D Lorenzo-Vivas E. Genomic Characterization of Murine Monocytes Reveals C/EBPbeta Transcription Factor Dependence of Ly6C(-) Cells. Immunity (2017) 46(5):849–62.e7. 10.1016/j.immuni.2017.04.018 28514690

[17] Li H van der Leun AM Yofe I Lubling Y Gelbard-Solodkin D van Akkooi ACJ. Dysfunctional CD8 T Cells Form a Proliferative, Dynamically Regulated Compartment Within Human Melanoma. Cell (2019) 176(4):775–89.e18. 10.1016/j.cell.2018.11.043 30595452 PMC7253294

[18] Welsh RM Bahl K Marshall HD Urban SL. Type 1 Interferons and Antiviral CD8 T-Cell Responses. PloS Pathog (2012) 8(1):e1002352. 10.1371/journal.ppat.1002352 22241987 PMC3252364

[19] Conroy H Marshall NA Mills KH. TLR Ligand Suppression or Enhancement of Treg Cells? A Double-Edged Sword in Immunity to Tumours. Oncogene (2008) 27(2):168–80. 10.1038/sj.onc.1210910 18176598

[20] Plitas G Konopacki C Wu K Bos PD Morrow M Putintseva EV. Regulatory T Cells Exhibit Distinct Features in Human Breast Cancer. Immunity (2016) 45(5):1122–34. 10.1016/j.immuni.2016.10.032 PMC5134901 27851913

[21] Marshall JD Fearon KL Higgins D Hessel EM Kanzler H Abbate C. Superior Activity of the Type C Class of ISS *In Vitro* and *In Vivo* Across Multiple Species. DNA Cell Biol (2005) 24(2):63–72. 10.1089/dna.2005.24.63 15699627

[22] Guilliams M Scott CL. Does Niche Competition Determine the Origin of Tissue-Resident Macrophages? Nat Rev Immunol (2017) 17(7):451–60. 10.1038/nri.2017.42 28461703

[23] den Haan JM Kraal G Bevan MJ. Cutting Edge: Lipopolysaccharide Induces IL-10-Producing Regulatory CD4+ T Cells That Suppress the CD8+ T Cell Response. J Immunol (2007) 178(9):5429–33. 10.4049/jimmunol.178.9.5429 PMC2776046 17442923

[24] Higgins SC Lavelle EC McCann C Keogh B McNeela E Byrne P. Toll-Like Receptor 4-Mediated Innate IL-10 Activates Antigen-Specific Regulatory T Cells and Confers Resistance to Bordetella Pertussis by Inhibiting Inflammatory Pathology. J Immunol (2003) 171(6):3119–27. 10.4049/jimmunol.171.6.3119 12960338

[25] Yoneyama M Kikuchi M Matsumoto K Imaizumi T Miyagishi M Taira K. Shared and Unique Functions of the DExD/H-Box Helicases RIG-I, MDA5, and LGP2 in Antiviral Innate Immunity. J Immunol (2005) 175(5):2851–8. 10.4049/jimmunol.175.5.2851 16116171

[26] Saito T Gale M Jr. Differential Recognition of Double-Stranded RNA by RIG-I-Like Receptors in Antiviral Immunity. J Exp Med (2008) 205(7):1523–7. 10.1084/jem.20081210 PMC2442628 18591413

[27] Castle JC Kreiter S Diekmann J Lower M van de Roemer N de Graaf J. Exploiting the Mutanome for Tumor Vaccination. Cancer Res (2012) 72(5):1081–91. 10.1158/0008-5472.CAN-11-3722 22237626

[28] Smyth MJ Ngiow SF Ribas A Teng MW. Combination Cancer Immunotherapies Tailored to the Tumour Microenvironment. Nat Rev Clin Oncol (2016) 13(3):143–58. 10.1038/nrclinonc.2015.209 26598942

