# Supplementary figures and images for: Intra-Tumoral Activation of Endosomal TLR Pathways Reveals a Distinct Role for TLR3 Agonist Dependent Type-1 Interferons in Shaping the Tumor Immune Microenvironment
Source: Front Oncol. 2021 Jul 26;11:711673. doi: 10.3389/fonc.2021.711673 (PMC8351420; doi:10.3389/fonc.2021.711673)

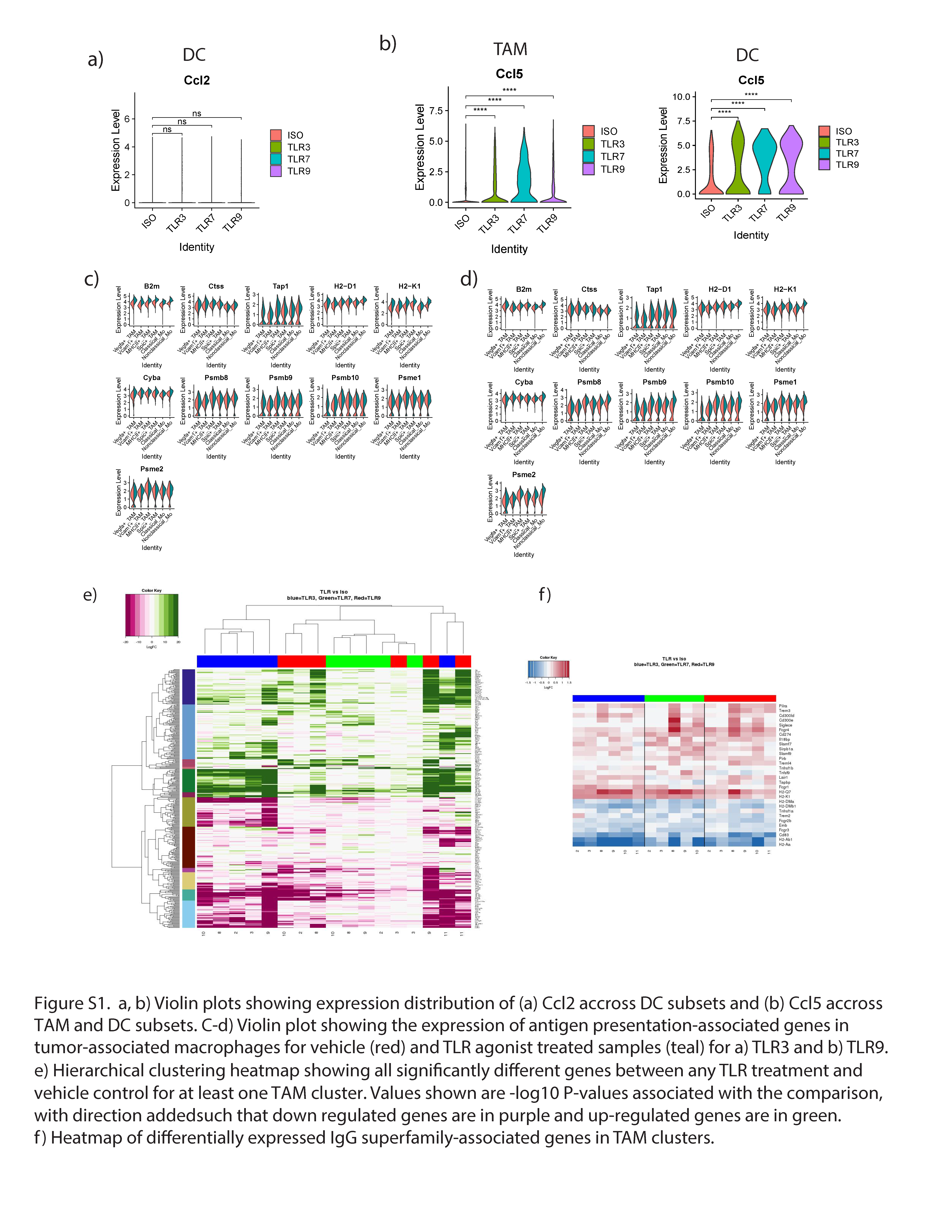

Supplement: Supplementary file 1 [file Image_1.tiff]

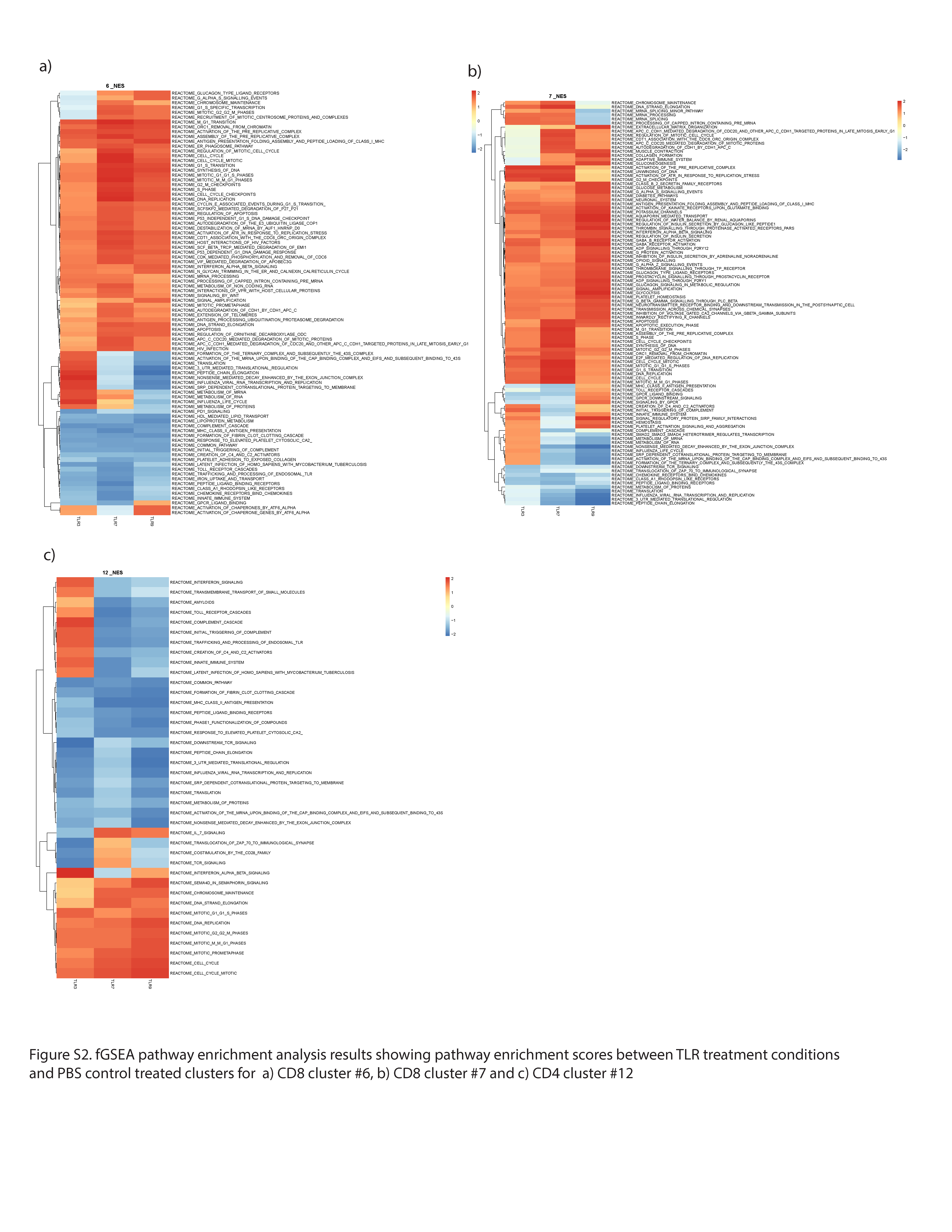

Supplement: Supplementary file 2 [file Image_2.tiff]
